# Developmental Trajectories of Associative Memory from Childhood to Adulthood: A Behavioral and Neuroimaging Study

**DOI:** 10.3389/fnbeh.2013.00126

**Published:** 2013-09-27

**Authors:** Bérengère Guillery-Girard, Sylvie Martins, Sebastien Deshayes, Lucie Hertz-Pannier, Catherine Chiron, Isabelle Jambaqué, Brigitte Landeau, Patrice Clochon, Gaël Chételat, Francis Eustache

**Affiliations:** ^1^INSERM, U1077, Caen, France; ^2^Université de Caen Basse-Normandie, UMR-S1077, Caen, France; ^3^Ecole Pratique des Hautes Etudes, UMR-S1077, Caen, France; ^4^CHU de Caen, UMR-S1077, Caen, France; ^5^INSERM, U663, Paris, France; ^6^Université Paris Descartes, Paris, France; ^7^CEA Saclay, Gif sur Yvette, France; ^8^SHFJ et Neurospin, I2BM, CEA, Paris, France; ^9^Unité de Neurochirurgie Pédiatrique, Fondation Ophtalmologique Adolphe de Rothschild, Paris, France

**Keywords:** episodic memory, associative memory, spatial memory, sequential memory, recollection, structural imaging

## Abstract

Episodic memory refers to the capacity to bind multimodal memories to constitute a unique personal event. Most developmental studies on episodic memory focused on one specific component, i.e., the core factual information. The present study examines the relevance of a novel episodic paradigm to assess its developmental trajectories in a more comprehensive way according to the type of association (item-feature, item-location, and item-sequence associations) with measures of both objective and subjective recollection. We conducted a behavioral study aimed at testing the effects of age in a large sample of 160 children, adolescents, and young adults (6–23 years old). We confronted the behavioral data to the neural correlates in a subgroup of 30 children using voxel-based morphometry. Behavioral data outlined differential developmental trajectories according to the type of association, with a continuous increase of factual associative memory efficiency until 10 years, a linear increase of performance in spatial associative memory that pursues until early adulthood and an abrupt increase in temporal associative memory efficiency between 9 and 10. Regarding recollection, measures showed a more pronounced enhancement from 9 to 10 years. Hence, behavioral data highlight a peculiar period in late childhood (8–10 years old) crucial for the developmental time course of episodic memory. Regarding structural data, we found that the improvement of associative memory efficiency was related to a decrease in gray matter volume in a large cerebral network including the dorsolateral and ventrolateral prefrontal cortex (and superior and anterior temporal regions), and the hippocampus bilaterally. These data suggest that multimodal integration would probably be related to the maturation of temporal regions and modulated by a fronto-parietal network. Besides, our findings emphasize the relevance of the present paradigm to assess episodic memory especially in the clinical setting.

## Introduction

Episodic memory refers to the most complex human memory system that emerges in early childhood. It requires both the individual’s self-awareness (i.e., autonoetic) of having personally experienced a past event while retrieving the overall phenomenological details (i.e., context or source) bound to that unique moment (which gives a peculiar vividness to the recall) and the ability to make sense of this recall for future experiences (Tulving, 2002). Thus, episodic memory processes rely on the binding of different types of associations, i.e., both within-domain associations such as inter-item associations (e.g., child and flower) and between-domain associations such as item-location associations (e.g., child and behind-window) (Mayes et al., 2007). These associations may be integrated into a single representation (e.g., the child standing behind the window) and may elicit a vivid sense of re-experiencing at retrieval (e.g., reliving the event with affective and perceptual information, i.e., recollection). Consequently, subjective recollection or “autonoetic” awareness would rely on a relational process (see Klein, 2013).

While several neurobehavioral models were applied to associative memory in adults (see Buchler et al., 2008), its maturation from childhood to early adulthood still needs to be explored in detail. *Within-domain* associative memory was tested in infancy in 9 months old children who were found to encode relations among two items (i.e., a face associated to a specific scenic background), and maintain this relational representation for a few seconds (Richmond and Nelson, 2009). This early developed capacity to bind information continues to improve from 4 to 6 (see Sluzenski et al., 2006; Lloyd et al., 2009 for the learning of item/background associations) and is supposed to reach adult efficiency in middle childhood. In a recent study, Thaler et al. (2013) suggested that long term associative memory, when assessed with an ordered repeated word list paradigm, may plateau from the age of 12 onward.

Other studies have focused on *between-domain* associations, either item/space or item/time respectively. As young as 2 years of age, toddlers are able to retain several item-location associations (Russell and Thompson, 2003) and performances still increase beyond the age of 10 (Barnfield, 1999; Gulya et al., 2002; Hund and Plumert, 2002). Processes of item-location memory may continue to refine until 18–20 years (Lorsbach and Reimer, 2005; see Pirogovsky et al., 2009 for odor-place associations). From 4 years on, children are able to encode temporal parameters that are knowledgeable, i.e., referring to a day. However, it is only later on, around 8 years old, that children can reliably localize multiple events extended into the past (Friedman and Lyon, 2005; Pathman et al., 2013). In sum, the developmental trajectories of associative memory seem to differ according to the component implicated (Picard et al., 2012). Within-domain associative memory dealing with factual information (item/background for instance) may plateau before between-domain associative memory. The maturation of these different types of associative memory may contribute to the development of subjective recollection. Studies that explored subjective experience with Remember/know paradigms reported slight or no modification of familiarity process with age contrary to subjective recollection (Billingsley et al., 2002; Brainerd et al., 2004; Piolino et al., 2007; Ghetti and Angelini, 2008). It is noteworthy that comparisons across developmental studies are challenging due to methodological issues with multiple paradigms and variables that may impact pediatrics behavioral data such as motivational, regulatory, and socio-educational factors.

Complex neurodevelopmental factors account for associative memory evolution from childhood to late adolescence, and can be studied by novel imaging techniques addressing various processes of cerebral maturation (Ghetti and Bunge, 2012). Looking at the cerebral maturation that supports associative memory enhancement from childhood to adolescence, two recent reviews focused on the role of both the prefrontal cortex (PFC) and the hippocampus in such associative processes (Ghetti and Bunge, 2012; Ofen et al., 2012). These two regions show distinct maturational time courses. Overall, PFC maturation appears more prolonged than in other regions (Gogtay et al., 2004). The protracted maturation of the dorsolateral PFC (Giedd, 2004) up to 20 years old seems to be associated with a progressive and late development of top-down attention modulation and strategic processes (Sander et al., 2012). The sparse structural studies that correlated children’s performance in standard memory tests with anatomical data provide further evidence that maturational changes in frontal regions [decrease in either cortical thickness (Sowell et al., 2001; Østby et al., 2012) or gray matter volume (Antshel et al., 2008)], are related to increasing memory efficiency in this developmental period. Developmental trends of the hippocampus and their relations with associative memory still need to be clarified.

Because associative memory is a defining feature of episodic memory, the understanding of associative memory development needs to take into consideration the type of associations in order to better describe children’s capacity to form and retrieve episodic memories. To date, no behavioral studies have explored the three associative domains (i.e., factual – WHAT, spatial – WHERE, and temporal – WHEN) with a single protocol in the same sample. Thus, the first goal of the present study is to describe the development of within-domain and inter-domain associative memory in a large sample of 160 healthy participants. We further examined our protocol’s reliance by confronting these behavioral results to structural imaging data collected in a subgroup of 30 children and adolescents to identify the regions that subserve episodic memory efficiency from childhood to young adulthood. Finally, to consider the possible influence of verbalization and retrieval processes on associative memory efficiency during development, we conducted additional correlational analyses between the 30 participants’ performance in verbal fluency and (1) behavioral data (the WHAT-WHERE-WHEN paradigm) and (2) volume of cerebral regions related to associative memory.

## Materials and Methods

### Participants

We conducted the behavioral study on participants aged from 6 to 23 years old recruited among several French Schools, High Schools, and Universities. Exclusion criteria were as follows: history of previous neurological disease, head trauma, current psychoactive medication, and learning disabilities. Families were given a comprehensive description of the research. We obtained written consent from parents of minors, in line with the guidelines of the relevant ethics committees. Informed consent was also obtained from participants over the age of 18. All participants completed the WHAT-WHERE-WHEN episodic memory paradigm (Guillery-Girard et al., 2010). One hundred children from 6 to 10 years (from childhood to adolescence; Mean = 100.7 ± 17.04 months, 51 females) and 60 adolescents and young adults from 12 to 23 years (from adolescence to adulthood; Mean = 194.30 ± 49.90 months, 29 females) were involved in the behavioral study. The children sample had been equally divided among five age groups (each group *n* = 20): 6 years old group (Mean = 77.1 ± 4 months, 11 females), 7 years old group (Mean = 88.7 ± 3.54 months, 12 females), 8 years old group (Mean = 109.5 ± 3.65 months, 12 females), 9 years old group (Mean = 113.2 ± 3.05 months, 7 females), and 10 years old group (Mean = 123.55 ± 3.27 months, 9 females). Similarly, the adolescent and adult sample had been equally divided among three age groups: 11–12 years old group (Mean = 142.55 ± 6.14 months, 10 females), 14–15 years old group (Mean = 180.58 ± 6.77 months, 12 females), and 20–23 years old group (Mean = 259.5 ± 12.86 months, 9 females).

Among these participants, 30 right-handed children and adolescents underwent a morphological MRI [age range: 79–180 months (6.6–15 years), Mean = 135.77 ± 28.19 months (11.31 ± 2.35 years), 13 females]. They additionally participated in various standard neuropsychological tests among which a verbal fluency test (i.e., this French task requires the participants to generate as many words as possible, firstly in a letter fluency condition and secondly, in a category fluency condition – 60 s are provided for each condition). All 30 children and adolescents accurately performed both the behavioral and the morphological MRI investigations on the same day.

#### Behavioral task

##### The “WHAT-WHERE-WHEN” paradigm

**Figure 1:**
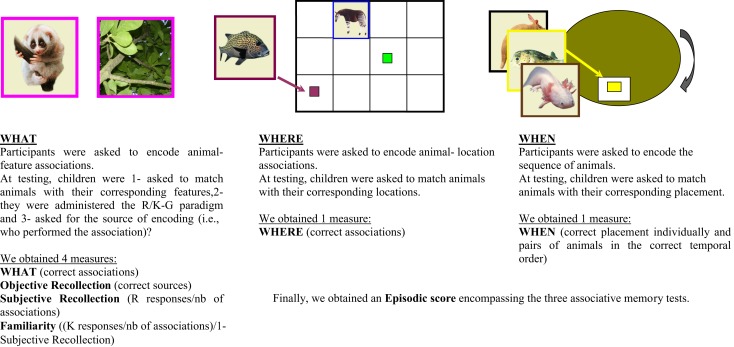
**«WHAT-WHERE-WHEN»paradigm**.

The WHAT task relied on within-domain factual associations and required participants to learn either 10 (for children aged 6–10 years) or 13 animal-feature associations (11–23) (Figure 1). Different list lengths were used to prevent possible ceiling and floor effects for the different age groups (Perez et al., 1998). During the encoding phase, children were shown photographs in a special book with the animal always displayed on the left-hand side and its feature on the right-hand side. Each double page was presented for 5 s (i.e., total encoding time of 50–65 s for the all set of associations). One third of the associations had already been matched and displayed in the book prior to the presentation; another third were to be matched by the experimenter; the remaining third were to be matched by the children themselves. That is, two thirds of the feature photos were laid out in front of the experimenter and the children, at their disposal for them to complete the associations. Color frames were used to emphasize each animal-feature association and to cue children with animal-feature relationship. For instance, photograph of the vicuna displayed in the book was surrounded by a green frame which indicated that its related feature (laid out in the middle of the features set) should be matched according to this color frame. The three different ways of matching the animal and its feature represented three different sources of encoding or “objective recollection” at retrieval. In contrast with intentional encoding of the animal-feature associations, source encoding was deliberately incidental, in order to approach ecological situations as closely as possible. At testing, the children were asked to match the animals with their corresponding features. Thus, the experimenter placed all the features in front of the children, minus their color frame, and provided the animal one at a time. Once, the children matched the feature with the current animal, the experimenter added a second copy of the chosen feature in the set of feature photographs available and removed the photographs of animal-feature association to avoid potential deductions. To assess the subjective recollection that accompanied the retrieval of each animal-feature association, the children were administered the Remember-Know paradigm. This paradigm was adapted for children by using a comic strip comprising three “smiley faces” representing “I remember,” “I know,” and “I guess” answers. Each type of response was carefully described in order to ensure that children understood every concept. Thus, “I guess” was to be chosen when they were not sure of their answer, while “I know” and “I remember” were to be chosen when children were quite certain. “I know” was then to indicate retrieval of an animal-feature association without any specific details about the encoded episode, “I remember” indicated retrieval of an animal-feature association accompanied by a mental journey back into the past allowing the encoded episode to be relived and phenomenological details retrieved. The difference between Remember and Know was extensively explained to children. To highlight the Remember answers we used a film/video metaphor (i.e., “you remember quite well that they go together because you can see them together like at the first time, as if you were watching a film in which you are the character”). To contrast this with the Know answers, we used a computer metaphor (i.e., “you know quite well that this picture goes with this picture here because they are stored together somewhere in your head, like if they were stored together into a computer”). Specific examples were used to contrast the types of information: their date of birth to refer to personal semantic information and/or the last dance show they attended to refer to personal episodic memories. To ensure children’s appropriate use of criteria, they were asked to reformulate the instructions. This procedure was adapted to each subject and repeated until the experimenter was confident about the child’s understanding of the Remember and Know judgments (see Picard et al., 2009 for a similar approach in autobiographical memory). Finally, for each association, objective recollection was assessed by asking the children to retrieve the corresponding source, in the shape of the person who performed the match (matched by the experimenter; matched by the child or matched prior to the presentation).

The WHERE task relied on between-domain spatial associations and is similar to the sub-test “Memory for designs” from the standardized battery NEPSY II (Korkman et al., 2007). Children were tested on their encoding of the exact location of each animal in an array (3 × 4 grid for children aged 6–10 years; 4 × 4 grid for children aged 11–18 years). They encoded the animal-location association by matching the colored frame surrounding the photograph of the animal to a colored marker in the center of each box of the grid. Animals were presented one at a time and the child was asked to place each animal into the correct location. The order of animals presentation was randomized across participants. Animals remained in view as the child proceeded through the task. Once all animals were placed, children were given 1 min to review the animal-location associations. In the test condition, children were again provided with the animals one at a time and were asked to place them in the array. In this condition there were no color cues. Again, animals were randomized across children.

The WHEN task investigated between-domain sequential associative memory. Children were asked to encode the animal-sequence association by placing the animals into slots in a wooden wheel. As the wheel was turned, only one slot was visible at a time. As in the other tasks, the animal-sequence association was determined by color cues that appeared in each slot. Each color corresponded to a specific animal. In the encoding condition, children were provided with the full set of animals and required to place them into the slots following the sequence determined by the color showing in the slot at each turn of the wheel. Once all animals were in place, the children were given one opportunity to review the overall sequence for a maximum of 1 min. In the test condition, children were again provided with the complete set of animals and required to retrieve the exact encoded sequence. Again, there were no color cues in this condition.

The measures derived from the WHAT-WHERE-WHEN test were then as follows:

(i) For the WHAT task: (1) total number of correct animal-feature associations (WHAT); (2) total number of sources that were correctly provided (Objective Recollection); (3) ratio of Remember judgments provided for every accurate association (Subjective Recollection, with Recollection = Remember responses/total number of associations); (4) familiarity is calculated in absence of recollection (as defined by Yonelinas and Jacoby, 1995) with Familiarity = *K*/1-R, where *K* = Know responses/total number of associations. This formula allows us to analyze separately Know from Recollection judgments.
(ii) For the WHERE task: total number of correct animal-location associations (WHERE).
(iii) For the WHEN task, we have taken into account both the correct placement of the animals individually and pairs of animals in the correct temporal order (see Riggins et al., 2009 for a similar approach). Thus one point was credited for each animal that was assigned to its rightful place in the sequence and 1/2 point was credited for each animals that accurately followed the original order but were inaccurately placed in the sequence [example A: animal one in first position, animal two in second position, animal three in third position = (1 + 1 + 1) = 4 points; example B: animal seven in first position, animal eight in second position, and animal nine in third position = (2 × 1/2) = 1 point]. The WHEN hits thus corresponds to the sum of placement and order in the sequence. We normalized all associative memory scores and Objective Recollection scores. This transformation of these possible heterogeneous raw scores into a common domain is needed prior to combining them into an episodic score (see below) and conducting parametric statistical analysis on normal distributions.

In order to assess overall episodic memory development, we computed a composite score obtained as follow: first, we summed the three *z*-scores (WHAT, WHERE, and WHEN) for each participant. This score is not quite a *z*-score, so it has been finally divided by the square root of the sum of the variance of the three subtests (which equals 3 × 1 since we used *z*-score) plus twice the sum of the covariance of the three subtests. As they were *z*-score, their covariances equal their correlation coefficients. For detail, see “Combining different scores from tests” in “Advanced Topics” section of the Psych Assessment website. This score assesses more specifically episodic memory since it encompasses three primary components of associative memory.

episodic score

$$\begin{array}{rcll} & & =\frac{Z_{\text{WHAT}}+Z_{\text{WHERE}}+Z_{\text{WHEN}}}{\sqrt{\left(3+2\times \left(r_{\text{WHATWHERE}}+r_{\text{WHATWHEN}}+r_{\text{WHEREWHEN}}\right)\right)}} & \end{array}$$

#### Imaging data acquisition

3D T1 images were acquired at the UMR663, INSERM, in the Service Hospitalier Frédéric Joliot (CEA-I2BM, Orsay, France), on a 1.5T MRI System (Signa, LX, GEMS, USA), with the following parameters: TR = 9.9 ms; TE = 2 ms; IR-Prep-time = 600 ms; flip angle = 10°; voxel size:0.9 mm × 9 mm × 1.2 mm, acquisition time: 7′56″.

#### Image processing

MRI data were segmented, normalized to a pediatric sample of the NIH (*N* = 324, age range = 4.5–18.5 years, Fonov et al., 2011), and modulated using the VBM5.1 toolbox (Ashburner and Friston, 2005) implemented in the Statistical Parametric Mapping 5 (SPM) software (Wellcome Trust Centre for Neuroimaging, London, UK) to obtain maps of local gray matter volume corrected for brain size. Finally, each image was smoothed with a Gaussian kernel (FWHM = 12 mm). Furthermore, we used a gray matter explicit mask in the voxelwise analyses so as to restrain the analyses to the gray matter. This mask was obtained by first averaging all segmented gray matter and white matter images, then thresholding the gray matter average to include voxels with a probability higher than 0.4 and the white matter average to include voxels with a probability higher than 0.2. Finally, the final GM mask used in the analyses was obtained by subtracting the WM mask from the GM mask.

#### Image statistical analysis

We analyzed age-related changes on brain morphometry using two statistical models entering either age (linear) or age and age2 (quadratic) as predictors using this fitting model: volume = *a*0 + *a*1score + *a*2score^2^ + ϵ where *a*0, *a*1, and *a*2 are polynomial parameters to be found, and error represents the residual error of the model (Büchel et al., 1998; Hu et al., 2013). Finally, we included sex as a regressor of non-interest. In a second set of analyses, each *z*-score (WHAT, WHERE, and WHEN) and the composite score referring to episodic memory were related to brain morphometry. These analyses were conducted first on the whole brain and second within the hippocampus only using a hippocampus delineated on a template. We performed the same regression analyses as before replacing age by the episodic score. Note that the predictor variables were first orthogonalized before being entered together in the quadratic models: age and age^2^ for age-related analyses, episodic and episodic score^2^ for brain-behavior relationships analyses. Because any relationship between brain volumes and memory could be driven by the common effect of age or sex, regression analyses with episodic memory were conducted with two regressors of non-interest (age and sex).

Two statistical thresholds were used, one for the whole brain and another for the hippocampus. For the whole brain where widespread effects were expected, we used a statistical threshold of uncorrected *p* < 0.001 and cluster extent *K* > 500 voxels. For analyses focusing on the hippocampus, a less stringent uncorrected *p*-value cut-off of *p* < 0.01 was applied with a cluster extent *K* > 100 voxels. In this latter condition, more localized effects were expected resulting from developmental differences along the longitudinal axis (DeMaster and Ghetti, 2013).

#### Complementary analyses

Finally, we conducted a last set of correlations between fluency *z*-score (which combines both letter and category fluency) and: (1) each *z*-score (WHAT, WHERE, and WHEN) and the composite score referring to episodic memory, (2) mean of gray matter volumes in clusters previously identified in brain-memory relationships analyses.

## Results

### Behavioral results

#### Associative memory

**Figure 2:**
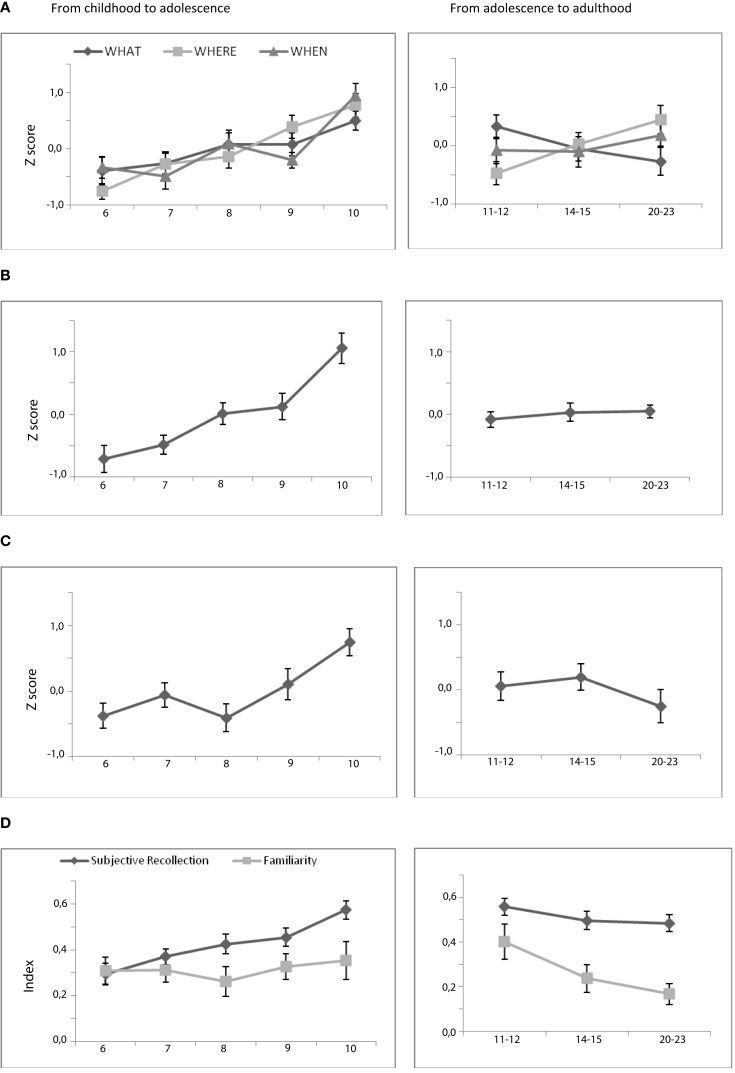
**Behavioral performances on the associative memory tasks**. **(A)** Mean performance of associative cued recall (hits) for each memory task as a function of age. **(B)** Mean episodic score. **(C)** Mean objective recollection. **(D)** Mean subjective recollection and familiarity index.

We performed one-way ANOVA for each task with age groups as between factor and Tukey’s adjustment for multiple comparisons (HSD). Those revealed three different developmental patterns (Figure 2).

##### Childhood to adolescence

First, the one-way ANOVA performed on the WHAT scores revealed a significant group effect [*F*(4, 95) = 2.54; *p* = 0.04] on performance associated with only one significant difference between 6- and 10-year-old groups (*p* = 0.04). These results illustrated a slight but significant increase from 6- to 10-year-olds. Second and considering the WHERE task, we observed a significant and linear increase in performance across age groups [*F*(4, 95) = 9.51; *p* < 0.001; 6 < 8 and 9 – 7 < 10 – 8 < 10-year-olds]. Finally, a significant group effect was also found for the WHEN task [*F*(4, 95) = 8.33; *p* < 0.001] with a ceiling effect until the age of 9 followed by a significant increase from 9- to 10-year-olds (*p* < 0.001, Figure 2A). This determining period also appeared in the analyses of the episodic scores [*F*(4, 95) = 14.56; *p* < 0.001] that combine the three associative memory tasks (Figure 2B). Tuckey *post hoc* tests revealed a slight increase from the age of 6 to 9 followed by a marked increase between 9- and 10-year-olds (6 < 7, 8, 9; 10 – 7 < 10 – 8 < 10 – 9 < 10).

##### Adolescence to adulthood

The one-way ANOVAs conducted on each of the associative score revealed a significant group effect only for WHERE’s performance [*F*(2, 57) = 4.74; *p* = 0.01; 11 – 12 < 21–24 age group]. No significant developmental difference was evident for neither the two other associative memory tasks nor the episodic score.

#### Objective and subjective recollection

##### Childhood to adolescence

Simple effects analysis conducted on the child groups indicated that the group effect observed on objective recollection [*F*(4, 95) = 5.10; *p* < 0.001] resulted from a significant increase from 8- to 10-year-olds (6, 7, 8 < 10, Figure 2C). In contrast, the significant group effect on subjective recollection [*F*(4, 95) = 6.44; *p* < 0.001] was related to a slight and linear increase of the ability of remembering (Figure 2D).

##### Adolescence to adulthood

Analysis conducted on the oldest age groups revealed that only the familiarity index decreased significantly from 11–12- to 21–23-year-olds [*F*(2, 57) = 3.56; *p* = 0.03; 11 – 12 > 21–24 age group].

### Imaging results

#### Age-related effects

**Figure 3:**
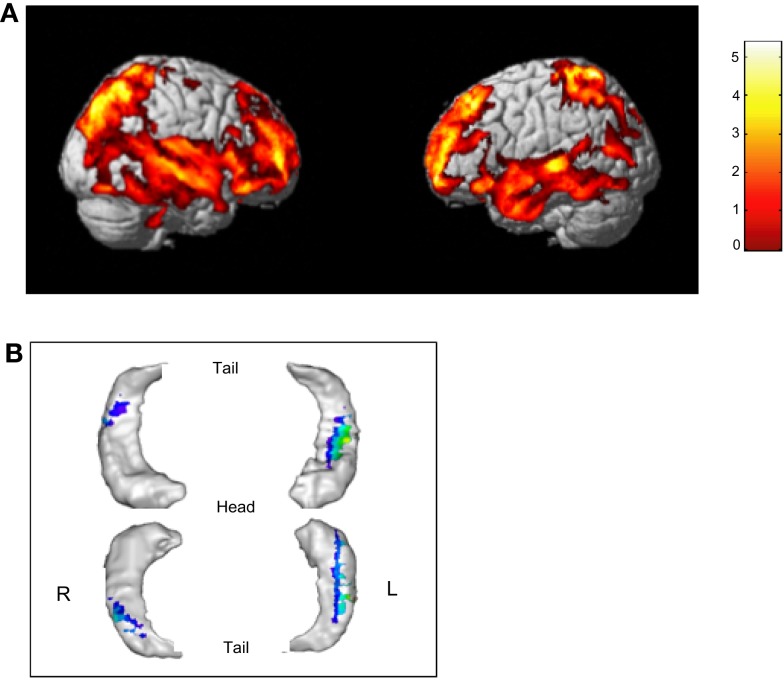
**Age-related effects on the brain**. **(A)** Age-related changes concern fronto-temporal and posterior parietal regions bilaterally (uncorrected *p* < 0.001, *K* > 500). **(B)** A significant reduction was found in the hippocampus bilaterally, with a more extended region in the left hippocampus (uncorrected *p* < 0.01, *K* > 100).

The linear regression analysis with total gray matter volume as the dependent variable and age as the predictor variable while controlling for the effect of sex, revealed a statistically significant volume decrease from 6 to 15 years (*R*^2^ = 0.449, *p* < 0.001). There was no significant contribution of age^2^, when this was included in the regression. Regional analyses revealed significant structural changes mainly in fronto-temporal regions but also involving posterior parietal regions (Figure 3). The regression analysis focused on the hippocampus, with the volume as the dependent variable and age as the predictor variable while controlling for the effect of sex, indicate a significant reduction in the volume of the hippocampal body bilaterally extending, in the left hemisphere, to the anterior part of the hippocampus.

#### Relationships between brain volume and memory performances

**Figure 4:**
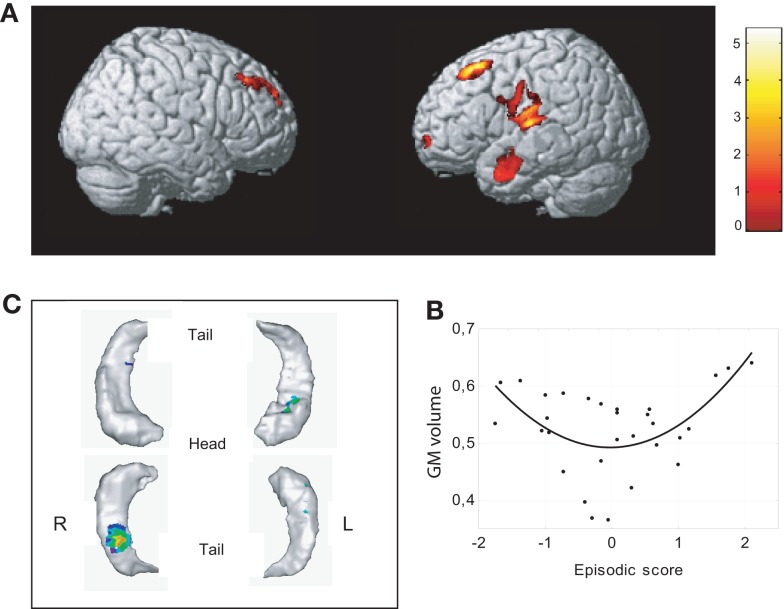
**Neural correlates of associative memory**. **(A)** Significant positive correlation (quadratic, U-shaped) with episodic score was found with the volume of gray matter in dorsolateral frontal regions bilaterally, the superior temporal cortex, the anterior middle temporal gyrus, and the ventrolateral prefrontal cortex (upper figure, uncorrected *p* < 0.001, *K* > 500). **(B)** Scatterplots of episodic effects are shown for regions identified in whole brain analyses. **(C)** Significant positive correlation (quadratic, U-shaped) was observed between memory and right hippocampal body and the anterior part of the left hippocampus (uncorrected *p* < 0.01, *K* > 100).

Our analyses failed to detect any statistically significant relationship between each *z*-score independently (i.e., WHAT, WHERE, and WHEN) and gray matter volume. On the contrary, in both whole brain and hippocampus template-based ROI, a quadratic positive relation (U-shaped) between volume and episodic score was found. Concerning the whole brain analysis, significant positive correlation with episodic memory was found with the volume of five brain areas in the right hemisphere (Figure 4; Table 1): (i) dorsolateral frontal regions, including the posterior part of the medial frontal gyrus, (ii) the superior temporal cortex encroaching both the transverse temporal gyrus around the lateral sulcus and the inferior parietal lobule, (iii) the anterior middle temporal gyrus, (iv) the ventrolateral prefrontal cortex (VLPFC) and, (v) the anterior part of dorsolateral PFC. Analyses performed on the hippocampus gray matter volume revealed significant relationships between memory efficiency and the body of the hippocampus on the right side and the anterior part of the hippocampus on the left side. Scatterplots of these effects showed that the enhancement of episodic performances was mainly associated with a decrease in mean volumes except for three participants with the highest scores.

**Table 1:** **Brain – associative memory relationships**.

Region		*x*	*y*	*z*	No. voxels	*z*
**WHOLE BRAIN (*p* **<** 0.001, UNCORRECTED, CLUSTER **>** 500)**						
L	Dorsolateral PF	−17	28	48	2985	4.34
L	Sup temporal	−54	−16	23	4278	4.18
L	Middle temporal	−65	−5	−22	2277	4.14
L	Ventrolateral PF	−38	63	−5	556	3.85
R	Dorsolateral PF	20	45	39	1646	3.71
**HIPPOCAMPUS (*p* **<** 0.01, UNCORRECTED, CLUSTER **>** 100)**						
L	Hippocampus	−34	−17	−20	598	2.92
R	Hippocampus	30	−34	−11	323	2.92

##### Complementary analyses

Correlational behavioral analyses revealed significant positive correlation between fluency *z*-score and each associative memory task (*p* = 0.004 WHAT; *p* = 0.026 WHERE; *p* = 0.003 WHEN) and the episodic composite score (*p* = 0.003), indicating that better efficiency in verbal fluency was associated with better performances in associative memory. Also, we observed a significant negative correlation between the fluency *z*-score and two clusters previously detected, i.e., dorsolateral frontal regions bilaterally and the superior temporal cortex. Thus, a decrease in volume in these three cortical regions was associated with increased efficiency in verbal fluency. These analyses did not detect any statistical correlation with the hippocampus.

## Discussion

The first aim of the present study was to describe the developmental trajectories of associative memory distinguishing within-domain (factual) and between-domain (spatial and temporal) associations by means of an original single paradigm. Results showed that these three types of associative memory follow distinct developmental trajectories: a slight but continuous increase of within-domain factual associative memory (WHAT), a linear increase of between-domain spatial associative memory (WHERE), and noticeable changes in between-domain temporal associative memory from 9 to 10 years (WHEN). Overall, the composite score that combines the three associative components of episodic memory (the “episodic score”) showed a slight but significant increase up to 9 years followed by a marked increase from 9 to 10. Regarding recollection, both objective (i.e., source memory) and subjective recollection improved with age with the most important effects from 8 to 10 years. Hence, these findings highlight major changes from 8–9 to 10 years. Thereafter, adolescence is characterized by slight changes, including enhancement of spatial associative memory and noticeable decrease in familiarity. Voxel-based morphometry suggests that episodic memory performance, which thus relies on remembering WHAT, WHERE, and WHEN components, is related to gray matter volume changes (i.e., following an inverted U-curve) in temporal regions including medial structures, prefrontal, and inferior parietal regions. Although these cortical regions and medial temporal structures have been previously described in functional studies, no such morphological data have been reported in developmental studies in relation with participants’ overall associative memory efficiency. Also, our results point out the relevance of using a single paradigm to assess associative memory, source memory, and recollection in order to get a more comprehensive picture of episodic memory development.

### Developmental trajectories of associative memory

Consistent with the developmental literature showing a major increase in memory efficiency between 6 and 10 years of age (Jambaqué et al., 1993; Vakil et al., 1998; Waber et al., 2007; Thaler et al., 2013), we found that these three types of associative memory (WHAT, WHERE, and WHEN) jointly improved in school-age children. The associative memory for within-domain features concerning the factual component follows a slight but significant increase from 6 to 10 years. Contrary to Sluzenski et al. (2006), we thus found that associative memory efficiency pursues its developmental course beyond the age of 6. Although the “WHAT” task shares some methodological characteristics with the animal-background associative paradigm proposed by these authors, i.e., to associate an animal with a specific feature, WHAT mainly differs on: (1) the use of a cued recall task known to be more age-sensitive than a recognition task (Picard et al., 2012), (2) the type of features that we designed (i.e., animals’ habitat or food), (3) the choice of unfamiliar animals to minimize putative prior semantic knowledge, and (4) the number of targets the participants were asked to remember. Furthermore, the regular increase in performance that we observed until 10 years was followed by a slight (though not significant) decrease during adolescence, which is consistent with previous studies using standard memory tests (Carey et al., 1980; Vakil et al., 1998; Waber et al., 2007). Accordingly, younger children may engage in piecemeal recording whereas older peers may deliberately organize the items to enhance memory efficiency. In adolescence, the enhancement of executive functions and verbal strategies (as assessed here by fluency tasks) may play a critical role in binding mechanisms (see by Rhodes et al., 2011; Picard et al., 2012 for recent data). The impact of the developing executive processes (in line with critical neural changes during adolescence; see Blakemore and Choudhury, 2006) on memory enhancement may explain, at least partially, the great variability in memory performance regularly discussed in the field of developmental neuropsychology (Picard et al., 2012).

In the present study, both spatial and temporal associative memories undergo specific developmental trajectories. Spatial associative memory follows a linear increase that continues from 10 onward. In contrast, we observed a large variability of temporal memory until 9 years followed by a marked increase from 9 to 10, plateauing afterward. These data are thus consistent with the idea that both spatial and temporal memory processes rely on distinct cognitive abilities subserved by distinguishable cerebral networks (Nyberg et al., 1996; Ekstrom and Bookheimer, 2007).

Spatial memory is not a unitary construct and implies peri-personal and extra-personal space processing, both being progressively bound together throughout normal development. These are linked to two different referential frames: allocentric and egocentric. In object-location memory tasks, both would interact with a probable superiority of the allocentric representation allowing individuals to code the location in a relational manner to the surrounding environment (Wang et al., 2005). Previous studies have shown that the type of cues used to remember a location changes from childhood to early adulthood (Bullens et al., 2010, 2011). Allocentric spatial abilities emerge around 2 years of age (Newcombe et al., 1998; Ribordy et al., 2013). However, when experimental designs increase in complexity, performances then depend on additional cognitive functions such as working memory (Lorsbach and Reimer, 2005), mental rotation, and the capacity to understand verbal instructions (Nardini et al., 2006). All these cognitive functions develop through childhood and adolescence and may contribute to the age-related effect on spatial processes observed in the present study from six to early adulthood.

Regarding temporal memory, we observe an abrupt increase in children’s performance at the age of nine followed by a plateau from 10 years onward. Memory for the temporal parameters of personal events or experimental items relies on several processes that refer to time (recency and frequency), location (labeling an event with external cues), and relative times of occurrence (sequential memory). The literature acknowledges that sequential memory needs to integrate both the relationship between an item and its position in the sequence and the relationship between two following items. To recollect the actual sequence, one needs to recreate the order in which stimuli were presented, that is to reorganize a set of items according to their temporal relationships. The ability to encode and retrieve sequences is thought to develop gradually from early childhood (McCormack and Hoerl, 1999) to adolescence in tandem with the development of language and organizational skills (Naito, 2003; Romine and Reynolds, 2004). Importantly, many authors support the view that the cognitive components of executive functions are fully efficient around the age of 12 (Anderson, 2002; Huizinga et al., 2006). Effective implementation of executive functions is essential for this kind of reconstructive process to be successful (Friedman and Lyon, 2005). Unexpectedly, we observe a slight, though not significant increase of performance during adolescence. Debriefing of the participants after the test allows us to hypothesize that this results from the particular design of the task itself. Indeed, many adolescents explained that they intentionally created a script on the bases of the animal orders (i.e., “when the urodel runs after the almiqui …”). Thus, the use of verbal strategies to encode the sequence in a chronological and meaningful order at this age may impact subsequent retrieval. Interestingly, this is in accordance with our additional analyses showing a significant correlation between verbal fluency efficiency and the WHEN.

Taken together, the above mentioned data suggest that associative episodic memory maturation depends on the type of information to be bound and that the overall processes may differentially contribute to episodic memory enhancement. Accordingly, the evolution of the combining episodic score through childhood tends to be non-linear prompted by the time course of temporal memory. Once more, the period of late childhood (9–10 years of age) is crucial in the developmental time course of episodic memory.

### Subjective and objective recollection

Objective and subjective recollection have been distinguished in functional (Spaniol et al., 2009 for review) and clinical studies (Duarte et al., 2008) in adults and have more recently encounter an increasing interest in the developmental literature (Ghetti and Angelini, 2008; Picard et al., 2009; Friedman et al., 2010).

In the present study, remember and familiarity judgments were equally distributed in the youngest group but appeared to follow two distinct time courses thereafter. Subjective recollection (as measured by the “remember” responses) showed significant enhancement up to 10 while familiarity remained unchanged during this period. Our findings concerning recollection are thus in accordance with recent data showing that 11 years old children were able to perform like adults (Rhodes et al., 2011). However, in the present study, familiarity judgment slightly decreased during adolescence contrasting with previous published behavioral data reporting no modification of familiarity process with age (Billingsley et al., 2002; Piolino et al., 2007). Yet, functional data sets as collected in Event Related Potential’s studies (Friedman et al., 2010) suggested that familiarity is unlikely to be driven by the exact same processes from childhood to late adolescence. The authors notably pointed out the relationship between familiarity and mid-frontal regions recruitment restricted to adolescents. Besides, these data argue for a comprehensive approach based on both neuroimaging and behavioral studies to understand age-related effects on episodic memory maturation.

We deliberately distinguished between subjective and objective recollection. A marked increase in “objective recollection” is reported from early childhood (4–6 years) onward (Perner and Ruffman, 1995; Welch-Ross, 1995) depending on factors such as distinctiveness, frequency, and retention interval (Parker, 1995; Ruffman et al., 2001). Recent data support the idea that objective and subjective recollection improve simultaneously from 6 to 18 years (Ghetti and Angelini, 2008; Ghetti et al., 2011). In the present study, objective (i.e., source memory) and subjective recollection followed slightly different developmental trajectories from 8 to 10 years and seemed more closely connected later on, i.e., from nine to adulthood. This developmental dissociation may be accounted for by difference in processes involved in recollection judgment with possibly preferred perceptual-based analysis in youngest participants (Ofen and Shing, 2013).

### Relationships between brain volume and memory efficiency

The present study pointed out the relationship between an increase in associative memory efficiency and a decrease in gray matter volume in a large cerebral network (including the dorsolateral and VLPFC, superior and anterior temporal regions) and the hippocampus bilaterally. In our sample, only three participants displayed a different pattern. As Shaw et al. (2006) argued in their study on global intellectual efficiency and cortical thickness, in which they reported different developmental trajectories of cortical thickness according to participants’ IQs, the variability in our sample may reflect different trajectories depending on the individual level of memory performance.

#### Frontal regions

Structural maturational changes in frontal lobes, decrease in either cortical thickness (Sowell et al., 2001) or volume (Antshel et al., 2008), are related with enhancement of episodic memory efficiency. Greater activation of lateral PFC was associated with an intentional encoding of scenes, subjective recollection (Ofen et al., 2007; Wendelken et al., 2011), retrieval suppression (Paz-Alonso et al., 2013), contextual memory (Ghetti et al., 2010) in children. However, no functional or structural study was conducted on associative multimodal memory in this population. The only structural study conducted in children and young adults so far indicates that thinner VLPFC may support more efficient relational encoding/retrieval processes of a complex Figure (Østby et al., 2012). In the present study, increased performances were related with volume reduction in the dorsolateral and the VLPFC. Interestingly, the reduction of DLPFC was also correlated with increased performances in verbal fluency in our sample, thus suggesting a possible contribution of executive functions to memory efficiency as measured by the WHAT-WHERE-WHEN paradigm.

#### Hippocampal structures

Only three other studies have investigated the relationships between memory and medial temporal lobe (Sowell et al., 2001) or hippocampal volume (Yurgelun-Todd et al., 2003; Østby et al., 2012). One reported a positive correlation with consolidation processes while the two other found negative correlations with retrieval performances. Otherwise, longitudinal studies focusing on age-related effects on gray matter volume revealed that the anterior part of the hippocampus decreases in volume from ages 4–25 years (Gogtay et al., 2006). In the present study, a slight decrease in hippocampal volume restricted to the right hippocampal body and the anterior part of the left hippocampus was related to an enhancement of episodic performances. Hence, our results suggest the involvement of a more anterior region in the right hippocampus that would be consistent with a progressive specialization evocated by DeMaster and Ghetti, 2013. However, further investigations are needed to understand the contribution of the hippocampus along its longitudinal axis to the development of episodic memory.

#### Temporo-parietal region

High-level associative areas allowing the integration of information from several sensory modalities undergo a protracted maturation up to early adulthood (Gogtay et al., 2006). Moreover, they support cognitive processes implicated in episodic memory, working memory updates associated with episodic retrieval (Borst and Anderson, 2013) or recollection (Yonelinas et al., 2005 in adults). The superior temporal gyrus is also implicated into self generation of verbal associations at encoding (Vannest et al., 2012 in adults). In the present study, we observed that the reduced volume in the superior temporal cortex extending to the inferior parietal lobule was related to episodic enhancement in children and adolescents. This region was not reported in previous structural studies but could support the generation of verbal strategies in our three associative tasks. The negative correlation between the volume reduction in the superior temporal cortex and the increase in verbal fluency performance would be consistent with this hypothesis. Moreover, is this cortical region involved in a larger fronto-parietal network including dorsolateral PFC that would implement cognitive control functions on memory functioning, and would support the progressive development of top-down attention modulation?

### Methodological considerations and future directions

Our findings bring interesting perspectives to assess episodic memory from childhood to adolescence in a more comprehensive way. To our knowledge, the present study is the first to address episodic memory maturational trajectories by investigating its main components within the same paradigm. It thus provides a more accurate picture of how episodic memory processes (i.e., binding of within/between-domain information, source memory, recollection, and familiarity) evolve.

By confronting behavioral results to structural data, we also describe the cortical regions that subserve episodic memory efficiency as a whole. Unfortunately, the present study failed to detect finer relationships between each component (factual, spatial, temporal, source memory, or recollection) and brain structures. Knowing the large inter-individual variability in both memory efficiency and cerebral maturation in children and adolescents, we assume that the lack of sensitivity in morphometric analyses could be overcome in a larger cohort.

Taken together, the behavioral and structural analyses questioned the implication of maturing executive processes that may explain, at least partially, the differential trajectories that we report according to the type of information to be encoded and recollected. This hypothesis needs to be directly addressed in a future study by using more dedicated executive tasks.

Besides its relevance to provide additional data to the research literature, the present paradigm is considered to be a sensitive and valid tool to assess episodic memory in the clinical setting. The WHAT-WHERE-WHEN paradigm has been administered to children and adolescents suffering from temporal lobe epilepsy and has successfully detected differential profiles of deficits according to epilepsy lateralization (Guillery-Girard et al., 2010). It thus can be helpful to reveal possible memory dissociations in young patients.

## Conclusion

Episodic memory refers to the capacity to bind different components into a single representation that will promote a vivid sense of re-experiencing at retrieval. Accordingly, our findings show the differential developmental trajectories of episodic memory processes in relationship with both cortical changes and neuropsychological factors such as verbal strategies and executive functions. Specifically, the present study suggests that the multimodal integration would be related to the maturation of temporal regions and may be modulated by a fronto-parietal network. Despite a limited sample for structural analyses, this study confirms the need to combine neuroimaging and behavioral data in order to better understand the developmental trajectory of episodic memory in a research setting, and to use a single paradigm to assess episodic memory as a whole in a clinical setting.

## Conflict of Interest Statement

The authors declare that the research was conducted in the absence of any commercial or financial relationships that could be construed as a potential conflict of interest.
